# Mol­ecular and crystal structure of methyl 4-methyl-2,2-dioxo-1*H*-2λ^6^,1-benzo­thia­zine-3-carboxyl­ate

**DOI:** 10.1107/S2056989018011362

**Published:** 2018-08-21

**Authors:** Svitlana Shishkina, Igor Ukrainets, Ganna Hamza, Lina Grinevich

**Affiliations:** a SSI "Institute for Single Crystals" National Academy of Sciences of Ukraine, 60, Nauky ave., Kharkiv 61001, Ukraine; b V.N. Karazin Kharkiv National University, 4 Svobody, Kharkiv 61077, Ukraine; c National University of Pharmacy, 4 Valentinovska Str., Kharkiv 61168, Ukraine

**Keywords:** 1,2-benzo­thia­zine derivatives, mol­ecular and crystal structure, hydrogen bonding, π-stacking inter­action

## Abstract

The title compound, C_11_H_11_NO_4_S, possesses weak analgesic properties and is a source compound for the synthesis of highly active analgesic and anti-inflammatory compounds. The benzo­thia­zine ring adopts a conformation intermediate between twist-boat and sofa. The ester substituent is turned towards the endocyclic double bond because of steric repulsion. In the crystal, the mol­ecules form columns along the [001] direction, bound by N—H⋯O hydrogen bonds and stacking inter­actions.

## Abstract 2

The mol­ecular and crystal structures of methyl 4-methyl-2,2-dioxo-1*H*-2λ^6^,1-benzo­thia­zine-3-carboxyl­ate, which possesses analgesic properties, have been determined

## Chemical context   

Methyl 4-methyl-2,2-dioxo-1*H*-2λ^6^,1-benzo­thia­zine-3-carb­oxyl­ate (**I**) displays moderate analgesic properties (Azotla-Cruz *et al.*, 2017 ▸) but has been used for the synthesis of highly active analgesic and anti-inflammatory compounds (Ukrainets *et al.*, 2018 ▸). Earlier it was shown (Ukrainets *et al.*, 2016*a* ▸,*b* ▸) that the biological properties of 2,1-benzo­thia­zine derivatives depend to a considerable degree on their mol­ecular and crystal structures. Thus knowledge of both the mol­ecular and crystal structures of **I** is very important.
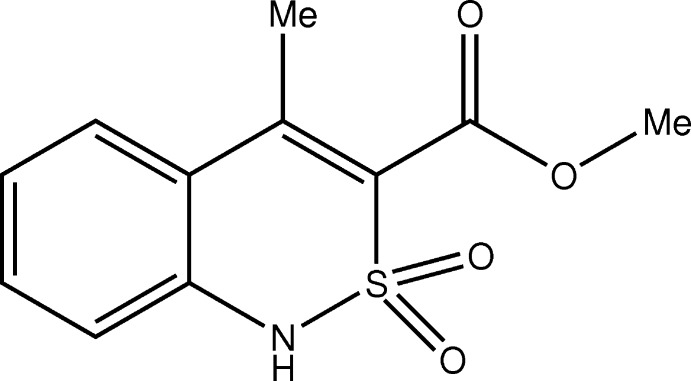


## Structural commentary   

**Figure 1:**
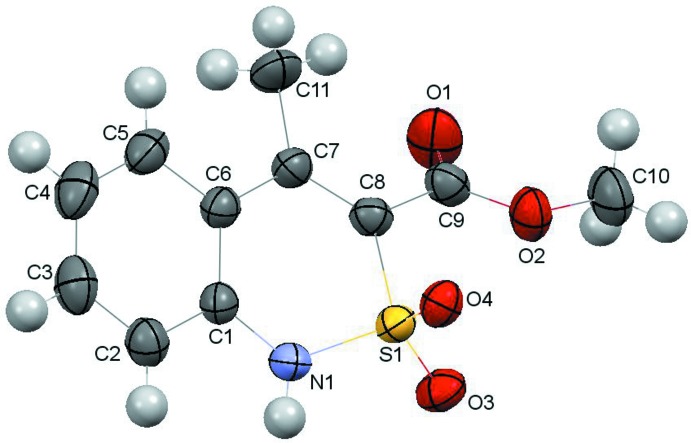
The mol­ecular structure of **I** with the atom labelling. Displacement ellipsoids are drawn at the 50% probability level.

The mol­ecular structure of the title compound is shown in Fig. 1 ▸. The di­hydro­thia­zine heterocycle adopts a twist-boat conformation with puckering parameters (Zefirov *et al.*, 1990 ▸) *S* = 0.57, Θ = 53.3°, Ψ = 25.2°. The S1 and C8 atoms deviate from the mean plane of the remaining ring atoms by 0.7941 (6) and 0.260 (2) Å, respectively. Some steric repulsion between the methyl substituent at the C7 atom and the ester group [the short intra­molecular contact C11⋯O1 is 2.986 (5) Å compared to the van der Waals radii sum of 3.00 Å (Zefirov, 1997 ▸)] is compensated for by the formation of the intra­molecular C11—H11*C*⋯O1 hydrogen bond (Table 1 ▸). As a result, the ester substituent is turned relative to the C7=C8 endocyclic double bond [C7=C8—C9—O1 torsion angle is −35.2 (5)°] and the C7=C8 [1.359 (4) Å] and C8—C9 [1.504 (3) Å] bonds are elongated compared to the standard values (Bürgi & Dunitz, 1994 ▸) of 1.326 and 1.455 Å, respect­ively. The methyl group of the ester substituent is in an anti-periplanar conformation relative to the C8—C9 bond [C8—C9—O2—C10 = 174.5 (2)°].

## Supra­molecular features   

**Figure 2:**
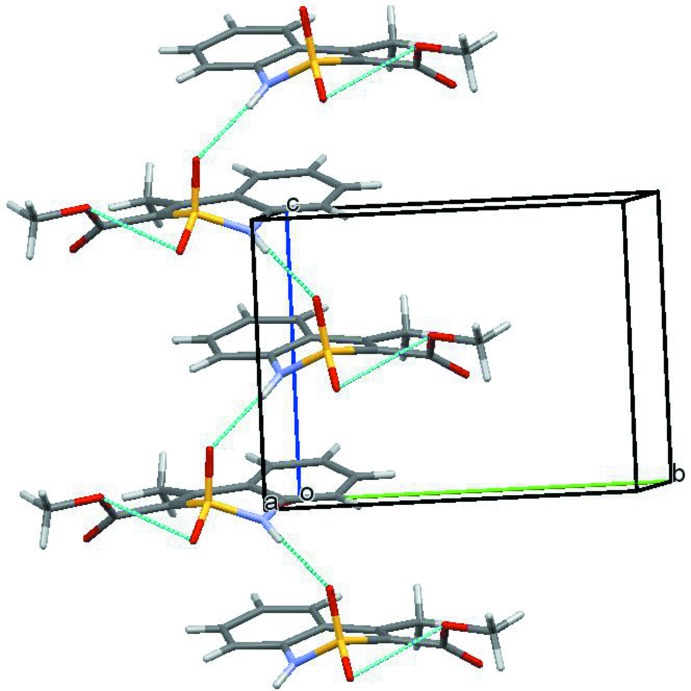
The packing showing columns of mol­ecules along the *c-*axis direction.

In the crystal, mol­ecules of **I** form columns along the [001] direction (Fig. 2 ▸). Neighboring mol­ecules within the column are linked by the N1—H1*N*⋯O4^i^ hydrogen bonds (Table 1 ▸) and π-stacking inter­actions with centroid–centroid diatances of 3.870 (2) Å. The columns are connected by weak C4—H4⋯O3^ii^ hydrogen bonds (Table 1 ▸).

## Database survey   

An search of the Cambridge Structural Database (Version 5.39, update February 2018; Groom *et al.*, 2016 ▸) revealed only three similar 1,2-benzo­thia­zine derivatives with a methyl substituent at the C7 atom (VAZQEV and VAZQIZ, Azotla-Cruz *et al.*, 2017 ▸; OWUQII, Azotla-Cruz *et al.*, 2016 ▸). All of these compounds are substituted at the nitro­gen atom and have very similar mol­ecular structures. The structure VAZQEV differs from others by the *trans*-orientation of the carbonyl group of the ester substituent relative to the endocyclic double bond.

### Synthesis and crystallization   

Methyl (chloro­sulfon­yl)acetate (1.90 g, 0.011 mol) was added dropwise with stirring to a solution of *ortho*-amino­aceto­phenone (1.35 g, 0.010 mol) and tri­ethyl­amine (1.54 mL, 0.011 mol) in CH_2_Cl_2_ (20 mL) and cooled to 268–273 K. After 10 h, water (50 mL) was added to the reaction mixture, which was then acidified to pH 4 with 1 *N* HCl and mixed thoroughly. The organic layer was separated off, dried over anhydrous CaCl_2_, and the solvent distilled (at reduced pressure at the end). The resulting anilide was subjected to heterocyclization without purification. A solution of sodium methyl­ate in anhydrous methanol [from metallic sodium (0.69 g, 0.030 mol) and absolute methanol (15 mL)], the mixture was boiled and then kept for 15 h at room temperature. The reaction mixture was diluted with cold water and acidified with 1 *N* HCl to pH 4. Finally, the solid ester, **I**, was separated by filtration, washed with water, and dried in air giving colourless block-shaped crystals, yield: 2.25 g (89%); m.p. 476–578 K (methanol); *R*_f =_ 0.37. ^1^H NMR (400 MHz, DMSO-*d*_6_): δ 11.84 (*br s*, 1H, NH), 7.79 (*d*, 1H, *J* = 7.6 Hz, H-5), 7.49 (*t*, 1H, *J* = 7.2 Hz, H-7), 7.22 (*t*, 1H, *J* = 7.6 Hz, H-6), 7.12 (*d*, 1H, *J* = 8.0 Hz, H-8), 3.84 (*s*, 3H, OCH_3_), 2.46 (*s*, 3H, 4-CH_3_, coincides with the signal of residual protons DMSO-*d*_6_). ^13^C-NMR (100 MHz, DMSO-*d*_6_ + CDCl_3_): δ 161.6 (C=O), 147.7, 138.2, 132.2, 127.4, 127.1, 123.0, 121.3, 118.8, 52.9 (OCH_3_), 17.5 (4-CH_3_). MS (*m*/*z*, %): 253 [*M*]^+^ (4.4), 252 [*M* − H]+ (1.5), 221 [*M* − CH_3_OH]^+^ (8.4), 195 (80.2), 119 (75.3), 103 (17.0), 93 (100), 92 (59.5), 77 (50.0). Analysis calculated for C_11_H_11_NO_4_S: C, 52.16; H, 4.38; N, 5.53; S 12.66%. Found: C, 52.07; H, 4.30; N, 5.46; S 12.72%.

## Refinement   

Crystal data, data collection and structure refinement details are summarized in Table 2 ▸. All of the H atoms were located in difference-Fourier maps. The N-bound H atoms were refined isotropically. The C-bound H atoms were included in calculated positions and treated as riding: C—H = 0.96 Å with *U*_iso_(H) =1.5*U*_eq_(C) for the methyl groups and C—H = 0.93 Å with *U*_iso_(H) = 1.2U_eq_(C) for all others.

## Supplementary Material

Crystal structure: contains datablock(s) I. DOI: 10.1107/S2056989018011362/zp2032sup1.cif

Structure factors: contains datablock(s) I. DOI: 10.1107/S2056989018011362/zp2032Isup2.hkl

Supporting information file. DOI: 10.1107/S2056989018011362/zp2032Isup3.cml

CCDC reference: 1861156

Additional supporting information:  crystallographic information; 3D view; checkCIF report

## Figures and Tables

**Table 1:** Hydrogen-bond geometry (Å, °)

*D*—H⋯*A*	*D*—H	H⋯*A*	*D*⋯*A*	*D*—H⋯*A*
C11—H11*C*⋯O1	0.96	2.24	2.986 (5)	133
N1—H1*N*⋯O4^i^	0.81 (4)	2.09 (4)	2.891 (3)	170 (4)
C4—H4⋯O3^ii^	0.93	2.55	3.427 (3)	158

Symmetry codes: (i) 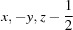; (ii) 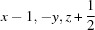.

**Table 2:** Experimental details

Crystal data	
Chemical formula	C_11_H_11_NO_4_S
*M*_r_	253.27
Crystal system, space group	Monoclinic, *P* *c*
Temperature (K)	293
*a*, *b*, *c* (Å)	7.8367 (3), 9.6842 (4), 7.5006 (4)
β (°)	93.468 (4)
*V* (Å^3^)	568.19 (4)
*Z*	2
Radiation type	Mo *K*α
μ (mm^−1^)	0.29
Crystal size (mm)	0.21 × 0.18 × 0.15

Data collection	
Diffractometer	Agilent Xcalibur Sapphire3
Absorption correction	Multi-scan (*CrysAlis RED*; Agilent, 2012 ▸)
*T*_min_, *T*_max_	0.809, 1.000
No. of measured, independent and observed [*I* > 2σ(*I*)] reflections	5509, 3068, 2803
*R*_int_	0.026
(sin θ/λ)_max_ (Å^−1^)	0.703

Refinement	
*R*[*F* ^2^ > 2σ(*F* ^2^)], *wR*(*F* ^2^), *S*	0.035, 0.085, 1.04
No. of reflections	3068
No. of parameters	160
No. of restraints	2
H-atom treatment	H atoms treated by a mixture of independent and constrained refinement
Δρ_max_, Δρ_min_ (e Å^−3^)	0.19, −0.21
Absolute structure	Flack *x* determined using 1127 quotients [(*I* ^+^)−(*I* ^−^)]/[(*I* ^+^)+(*I* ^−^)] (Parsons et al., 2013 ▸)
Absolute structure parameter	0.04 (5)

Computer programs: *CrysAlis CCD* and *CrysAlis RED* (Agilent, 2012 ▸), *SHELXS2014/7* (Sheldrick, 2008 ▸), *SHELXL2014/7* (Sheldrick, 2015 ▸) and *Mercury* (Macrae *et al.*, 2008 ▸).

## supplementary crystallographic information

### Crystal data

**Table:**

C_11_H_11_NO_4_S	*F*(000) = 264
*M**_r_* = 253.27	*D*_x_ = 1.480 Mg m^−^^3^
Monoclinic, *P**c*	Mo *K*α radiation, λ = 0.71073 Å
*a* = 7.8367 (3) Å	Cell parameters from 2089 reflections
*b* = 9.6842 (4) Å	θ = 4.2–30.6°
*c* = 7.5006 (4) Å	µ = 0.29 mm^−^^1^
β = 93.468 (4)°	*T* = 293 K
*V* = 568.19 (4) Å^3^	Block, colourless
*Z* = 2	0.21 × 0.18 × 0.15 mm

### Data collection

**Table:**

Agilent Xcalibur Sapphire3 diffractometer	3068 independent reflections
Radiation source: Enhance (Mo) X-ray Source	2803 reflections with *I* > 2σ(*I*)
Detector resolution: 16.1827 pixels mm^-1^	*R*_int_ = 0.026
ω–scans	θ_max_ = 30.0°, θ_min_ = 3.4°
Absorption correction: multi-scan (CrysAlis RED; Agilent, 2012)	*h* = −10→11
*T*_min_ = 0.809, *T*_max_ = 1.000	*k* = −9→13
5509 measured reflections	*l* = −10→10

### Refinement

**Table:**

Refinement on *F*^2^	Secondary atom site location: difference Fourier map
Least-squares matrix: full	Hydrogen site location: difference Fourier map
*R*[*F*^2^ > 2σ(*F*^2^)] = 0.035	H atoms treated by a mixture of independent and constrained refinement
*wR*(*F*^2^) = 0.085	*w* = 1/[σ^2^(*F*_o_^2^) + (0.0399*P*)^2^] where *P* = (*F*_o_^2^ + 2*F*_c_^2^)/3
*S* = 1.04	(Δ/σ)_max_ < 0.001
3068 reflections	Δρ_max_ = 0.19 e Å^−^^3^
160 parameters	Δρ_min_ = −0.21 e Å^−^^3^
2 restraints	Absolute structure: Flack *x* determined using 1127 quotients [(*I*^+^)-(*I*^-^)]/[(*I*^+^)+(*I*^-^)] (Parsons et al., 2013)
Primary atom site location: structure-invariant direct methods	Absolute structure parameter: 0.04 (5)

### Special details

**Table:**

Geometry. All esds (except the esd in the dihedral angle between two l.s. planes) are estimated using the full covariance matrix. The cell esds are taken into account individually in the estimation of esds in distances, angles and torsion angles; correlations between esds in cell parameters are only used when they are defined by crystal symmetry. An approximate (isotropic) treatment of cell esds is used for estimating esds involving l.s. planes.

### Fractional atomic coordinates and isotropic or equivalent isotropic displacement parameters (Å^2^)

**Table:**

	*x*	*y*	*z*	*U*_iso_*/*U*_eq_	
S1	0.82278 (8)	0.16925 (5)	0.52933 (8)	0.03401 (14)	
O1	0.6143 (4)	0.5195 (2)	0.4202 (4)	0.0679 (7)	
O2	0.8581 (3)	0.4555 (2)	0.5668 (3)	0.0514 (5)	
O3	0.9468 (3)	0.2019 (2)	0.4041 (3)	0.0477 (5)	
O4	0.8846 (2)	0.15240 (18)	0.7126 (2)	0.0416 (4)	
N1	0.7250 (3)	0.0322 (2)	0.4558 (3)	0.0420 (5)	
H1N	0.780 (5)	−0.012 (4)	0.388 (5)	0.064 (11)*	
C1	0.5686 (3)	−0.0083 (2)	0.5189 (3)	0.0364 (5)	
C2	0.5276 (4)	−0.1480 (3)	0.5226 (4)	0.0459 (6)	
H2	0.6049	−0.2136	0.4864	0.055*	
C3	0.3720 (5)	−0.1882 (3)	0.5802 (4)	0.0558 (8)	
H3	0.3433	−0.2814	0.5814	0.067*	
C4	0.2584 (4)	−0.0915 (4)	0.6361 (4)	0.0570 (8)	
H4	0.1550	−0.1198	0.6786	0.068*	
C5	0.2967 (4)	0.0469 (3)	0.6297 (4)	0.0469 (6)	
H5	0.2174	0.1112	0.6652	0.056*	
C6	0.4538 (3)	0.0927 (3)	0.5705 (3)	0.0374 (5)	
C7	0.4909 (3)	0.2408 (3)	0.5508 (3)	0.0384 (5)	
C8	0.6512 (3)	0.2858 (3)	0.5222 (3)	0.0365 (5)	
C9	0.7020 (4)	0.4337 (3)	0.4961 (4)	0.0431 (6)	
C10	0.9327 (5)	0.5898 (3)	0.5392 (5)	0.0634 (9)	
H10A	1.0462	0.5924	0.5949	0.095*	
H10B	0.8640	0.6595	0.5908	0.095*	
H10C	0.9375	0.6066	0.4134	0.095*	
C11	0.3470 (5)	0.3403 (3)	0.5685 (6)	0.0599 (10)	
H11A	0.3055	0.3331	0.6860	0.090*	
H11B	0.2562	0.3192	0.4811	0.090*	
H11C	0.3869	0.4326	0.5498	0.090*	

### Atomic displacement parameters (Å^2^)

**Table:**

	*U*^11^	*U*^22^	*U*^33^	*U*^12^	*U*^13^	*U*^23^
S1	0.0300 (3)	0.0345 (2)	0.0379 (3)	0.0013 (2)	0.0052 (2)	0.0017 (2)
O1	0.0679 (16)	0.0430 (11)	0.0907 (18)	0.0062 (10)	−0.0134 (14)	0.0153 (11)
O2	0.0499 (13)	0.0386 (9)	0.0646 (14)	−0.0088 (9)	−0.0041 (10)	0.0034 (8)
O3	0.0423 (11)	0.0504 (11)	0.0519 (12)	0.0000 (9)	0.0161 (9)	0.0048 (9)
O4	0.0372 (10)	0.0453 (10)	0.0421 (11)	−0.0009 (7)	−0.0014 (8)	0.0061 (7)
N1	0.0383 (12)	0.0385 (11)	0.0505 (13)	−0.0010 (9)	0.0116 (10)	−0.0106 (9)
C1	0.0333 (12)	0.0388 (12)	0.0367 (13)	−0.0032 (10)	0.0000 (10)	−0.0034 (9)
C2	0.0472 (16)	0.0391 (13)	0.0508 (16)	−0.0035 (11)	−0.0007 (13)	0.0006 (11)
C3	0.059 (2)	0.0488 (16)	0.059 (2)	−0.0186 (14)	0.0022 (15)	0.0046 (12)
C4	0.0461 (17)	0.072 (2)	0.0530 (17)	−0.0189 (15)	0.0062 (13)	0.0059 (15)
C5	0.0349 (13)	0.0617 (17)	0.0444 (15)	−0.0021 (12)	0.0040 (12)	−0.0013 (12)
C6	0.0323 (12)	0.0426 (13)	0.0369 (12)	0.0005 (10)	−0.0004 (9)	−0.0029 (9)
C7	0.0328 (12)	0.0405 (13)	0.0416 (13)	0.0036 (10)	−0.0010 (10)	−0.0047 (10)
C8	0.0370 (13)	0.0343 (11)	0.0383 (13)	0.0052 (9)	0.0010 (10)	−0.0012 (9)
C9	0.0474 (15)	0.0347 (12)	0.0469 (16)	0.0045 (11)	−0.0004 (13)	−0.0027 (10)
C10	0.069 (2)	0.0425 (17)	0.078 (2)	−0.0173 (15)	0.0017 (18)	−0.0029 (15)
C11	0.0372 (17)	0.0518 (15)	0.091 (3)	0.0120 (13)	0.0055 (18)	−0.0072 (15)

### Geometric parameters (Å, º)

**Table:**

S1—O3	1.4271 (19)	C1—C6	1.400 (3)
S1—O4	1.4391 (19)	C2—C3	1.375 (5)
S1—N1	1.613 (2)	C3—C4	1.375 (5)
S1—C8	1.754 (3)	C4—C5	1.375 (4)
O1—C9	1.199 (4)	C5—C6	1.406 (4)
O2—C9	1.321 (4)	C6—C7	1.472 (4)
O2—C10	1.446 (4)	C7—C8	1.359 (4)
N1—C1	1.397 (3)	C7—C11	1.496 (4)
C1—C2	1.391 (3)	C8—C9	1.503 (4)
			
O3—S1—O4	116.79 (13)	C4—C5—C6	121.1 (3)
O3—S1—N1	106.58 (13)	C1—C6—C5	117.2 (2)
O4—S1—N1	111.04 (12)	C1—C6—C7	121.3 (2)
O3—S1—C8	112.85 (12)	C5—C6—C7	121.4 (3)
O4—S1—C8	108.38 (12)	C8—C7—C6	121.2 (2)
N1—S1—C8	99.88 (13)	C8—C7—C11	121.1 (3)
C9—O2—C10	117.3 (2)	C6—C7—C11	117.7 (2)
C1—N1—S1	121.58 (18)	C7—C8—C9	125.5 (2)
C2—C1—N1	119.2 (2)	C7—C8—S1	120.2 (2)
C2—C1—C6	121.4 (2)	C9—C8—S1	114.1 (2)
N1—C1—C6	119.3 (2)	O1—C9—O2	124.7 (3)
C3—C2—C1	119.5 (3)	O1—C9—C8	125.0 (3)
C2—C3—C4	120.4 (3)	O2—C9—C8	110.3 (2)
C5—C4—C3	120.5 (3)		
			
O3—S1—N1—C1	−163.3 (2)	C1—C6—C7—C11	166.1 (3)
O4—S1—N1—C1	68.5 (2)	C5—C6—C7—C11	−9.2 (4)
C8—S1—N1—C1	−45.7 (2)	C6—C7—C8—C9	178.5 (2)
S1—N1—C1—C2	−149.9 (2)	C11—C7—C8—C9	−3.3 (4)
S1—N1—C1—C6	32.6 (3)	C6—C7—C8—S1	−6.1 (3)
N1—C1—C2—C3	−178.3 (3)	C11—C7—C8—S1	172.1 (2)
C6—C1—C2—C3	−0.8 (4)	O3—S1—C8—C7	145.0 (2)
C1—C2—C3—C4	−0.9 (5)	O4—S1—C8—C7	−84.1 (2)
C2—C3—C4—C5	2.1 (5)	N1—S1—C8—C7	32.2 (2)
C3—C4—C5—C6	−1.6 (4)	O3—S1—C8—C9	−39.2 (2)
C2—C1—C6—C5	1.3 (4)	O4—S1—C8—C9	91.8 (2)
N1—C1—C6—C5	178.8 (2)	N1—S1—C8—C9	−151.97 (19)
C2—C1—C6—C7	−174.3 (2)	C10—O2—C9—O1	−4.8 (5)
N1—C1—C6—C7	3.2 (4)	C10—O2—C9—C8	174.5 (2)
C4—C5—C6—C1	−0.1 (4)	C7—C8—C9—O1	−35.2 (5)
C4—C5—C6—C7	175.5 (2)	S1—C8—C9—O1	149.2 (3)
C1—C6—C7—C8	−15.6 (4)	C7—C8—C9—O2	145.5 (3)
C5—C6—C7—C8	169.1 (3)	S1—C8—C9—O2	−30.1 (3)

### Hydrogen-bond geometry (Å, º)

**Table:**

*D*—H···*A*	*D*—H	H···*A*	*D*···*A*	*D*—H···*A*
C11—H11*C*···O1	0.96	2.24	2.986 (5)	133
N1—H1*N*···O4^i^	0.81 (4)	2.09 (4)	2.891 (3)	170 (4)
C4—H4···O3^ii^	0.93	2.55	3.427 (3)	158

Symmetry codes: (i) *x*, −*y*, *z*−1/2; (ii) *x*−1, −*y*, *z*+1/2.
